# Mechanism of insulin resistance in a rat model of kidney disease and the risk of developing type 2 diabetes

**DOI:** 10.1371/journal.pone.0176650

**Published:** 2017-05-01

**Authors:** François Dion, Christopher Dumayne, Nathalie Henley, Stéphanie Beauchemin, Edward B. Arias, François A. Leblond, Sylvie Lesage, Stéphane Lefrançois, Gregory D. Cartee, Vincent Pichette

**Affiliations:** 1 Centre de recherche de l’Hôpital Maisonneuve-Rosemont, Faculté de Médecine, Centre affilié à l’Université de Montréal, Montréal, Québec, Canada; 2 Département de pharmacologie, Faculté de Médecine, Université de Montréal, Montréal, Québec, Canada; 3 School of Kinesiology, University of Michigan, Ann Arbor, Michigan, United States of America; 4 Département de microbiologie, infectiologie et immunologie, Faculté de Médecine, Université de Montréal, Montréal, Québec, Canada; 5 Centre INRS-Institut Armand-Frappier, Institut National de la Recherche Scientifique, Laval, Québec, Canada; 6 Department of Anatomy and Cell Biology, McGill University, Montreal, Quebec, Canada; Tohoku University, JAPAN

## Abstract

Chronic kidney disease is associated with homeostatic imbalances such as insulin resistance. However, the underlying mechanisms leading to these imbalances and whether they promote the development of type 2 diabetes is unknown. The effect of chronic kidney disease on insulin resistance was studied on two different rat strains. First, in a 5/6^th^ nephrectomised Sprague-Dawley rat model of chronic kidney disease, we observed a correlation between the severity of chronic kidney disease and hyperglycemia as evaluated by serum fructosamine levels (p<0.0001). Further, glucose tolerance tests indicated an increase of 25% in glycemia in chronic kidney disease rats (p<0.0001) as compared to controls whereas insulin levels remained unchanged. We also observed modulation of glucose transporters expression in several tissues such as the liver (decrease of ≈40%, p≤0.01) and muscles (decrease of ≈29%, p≤0.05). Despite a significant reduction of ≈37% in insulin-dependent glucose uptake in the muscles of chronic kidney disease rats (p<0.0001), the development of type 2 diabetes was never observed. Second, in a rat model of metabolic syndrome (Zucker Lepr^fa/fa^), chronic kidney disease caused a 50% increased fasting hyperglycemia (p<0.0001) and an exacerbated glycemic response (p<0.0001) during glucose challenge. Similar modulations of glucose transporters expression and glucose uptake were observed in the two models. However, 30% (p<0.05) of chronic kidney disease Zucker rats developed characteristics of type 2 diabetes. Thus, our results suggest that downregulation of GLUT4 in skeletal muscle may be associated with insulin resistance in chronic kidney disease and could lead to type 2 diabetes in predisposed animals.

## Introduction

The prevalence of chronic kidney disease (CKD) is steadily increasing [1], and its metabolic and hormonal complications have been the subject of many studies [2, 3]. In particular, type 2 diabetes (T2D), a leading cause of end-stage renal disease (ESRD), is an important and growing health problem [4, 5]. The convergence of these two conditions could lead to mutual exacerbation and worsening of patient health [6, 7].

Glucose intolerance is a common finding in patients and animals affected by renal impairment [8, 9]. Numerous studies suggest that peripheral insulin resistance and/or impaired insulin secretion are causative factors for diminished carbohydrate metabolism [10–13]. As insulin resistance in CKD is associated with diabetes, kidney disease could also lead to a deterioration of insulin sensitivity [11, 14]. The morbidity and mortality associated with CKD are predominantly attributable to cardiovascular complications, as more than half of the reported cases of death in patients with ESRD are linked to heart disease [15, 16]. Consequently, given that insulin resistance may contribute to the development of cardiovascular disease, insulin resistance is an important target to prevent such complications in patients with CKD.

Although insulin resistance in CKD is usually regarded as a post-receptor defect [16, 17], the mechanisms underlying this disorder are still poorly understood. Several lines of emerging evidence have demonstrated that CKD leads to modulation of the expression and activity of several drug transporters including those of the *ATP-Binding Cassette* and *Solute Carriers* families in the liver, intestine, kidneys and brain [18–20]. It therefore seems plausible that glucose transporters, members of the *Solute Carriers* family, may also be altered. To date, however, there is limited and conflicting data concerning alterations in the expression of glucose transporters in CKD and their role in insulin resistance [17, 21–23].

The purpose of this study was to determine whether: 1) CKD leads to insulin resistance in a nephrectomised rat model of CKD, 2) CKD modulates key transporters involved in glucose homeostasis which could ultimately lead to insulin resistance, 3) CKD is associated with impaired glucose uptake in insulin-sensitive organs and 4) insulin resistance in CKD leads to T2D. To address these aims, we used the 5/6th nephrectomy model to induce CKD in both Sprague-Dawley (SD) and Zucker Lepr^fa/fa^ rats, where the latter rat strain present a metabolic syndrome and, therefore, exhibit insulin resistance without developing T2D. These studies allowed us to evaluate the potential of kidney disease to precipitate the onset of T2D in predisposed patients.

## Materials and methods

### Ethical statement

All experiments were conducted according to the Canadian Council on Animal Care guidelines for the care and use of laboratory animals and were specifically approved by our local animal care committee (Comité de protection des animaux du Centre de recherche de l'Hôpital Maisonneuve-Rosemont; Protocol #2015–26). Isoflurane was used for anaesthesia during surgeries and all precautions were taken to minimize discomfort. At the end of the study, rats were euthanized by decapitation to avoid pharmacological interference with experiments.

### Animal models

Male Sprague-Dawley (SD) rats (Charles River Laboratories, St-Constant, QC, Canada), weighing 176 to 225 g, 6 to 8 weeks of age, and Zucker ZUC-Lepr^fa/fa^ rats (Charles River Laboratories, Raleigh, NC), weighing 180-200g, 6 weeks of age, were housed in the animal facility at the Hôpital Maisonneuve-Rosemont Research Center and were provided with Harlan Teklad rodent diet #2014 (Envigo, Lachine, QC, Canada) and water *ad libitum*.

### Surgery and experimental protocol

CKD was induced by a two-stage 5/6 nephrectomy as previously described [24]. A timeline of the experimental procedures is provided in supporting information S1 Fig. Chronic kidney disease (CKD) and control (CTL) rats were matched according to their weight and age at the start of the experiment. CKD rats underwent a 2/3 nephrectomy of the left kidney (Day 0) and 7 days later, a right total nephrectomy. CTL rats underwent two sham laparotomies. The amount of chow fed to control pair-fed rats was adjusted daily, based on the amount of chow that the CKD rats ate the day before. After Day 21, rats were monitored once a week for glycosuria. At Day 41, rats were housed in metabolic cages and urine was collected for 24 hours to determine creatinine clearance. Rats were sacrificed at Day 42 for organ and blood collection, unless stated otherwise. Liver, kidneys, *Biceps Femoris* and epididymal white adipose tissue were immediately excised, rinsed in phosphate buffered saline (PBS) and flash-frozen in liquid nitrogen. Blood was collected for further biochemical characterization. Rats used for glycosuria assessment were kept alive for up to 15 weeks. All days are calculated from 1^st^ nephrectomy. Rats with a fasting glycemia over 11mmol/L, with the presence of glycosuria as defined below, were defined as T2D.

### Biochemical parameters

Serum creatinine, urea, fructosamine and urinary creatinine were analyzed on an ARCHITECT c16000 Clinical Chemistry Analyzer (Abbott Diagnostics, Lake Forest, IL, USA). Glycemia was measured using an AlphaTRAK blood glucose monitoring system (Abbott Laboratories, North Chicago, IL, USA). Serum insulin was assayed by a Rat ultrasensitive ELISA kit (ALPCO, Salem, NH, USA). The presence of glycosuria was defined by a positive Diastix^™^ (Bayer Corp. Diagnostics, Tarrytown, NY, USA) test (>14mmol/L) on two consecutive mornings.

### Intraperitoneal glucose tolerance test

An intraperitoneal glucose tolerance test (IPGTT) was performed 4 weeks after the first nephrectomy (Day 28). After an overnight fast, rats were injected with an intraperitoneal bolus of D-glucose (BDH, Westchester, PA) (2g/kg of body weight for SD rats [25, 26] and 1.5g/kg for Zucker rats [26, 27]). Blood samples (200μl) were collected from the saphenous vein immediately before and 15, 30, 60, 90, and 120 minutes after glucose administration. Great care was taken to minimize stress in animals during blood retrieval. Glycemia was measured using an AlphaTRAK blood glucose monitoring system. Serum insulin levels were determined at each time point as described above, according to manufacturer’s protocol.

### *Ex vivo* glucose uptake in muscles

The glucose uptake capacity of two skeletal muscles, *soleus* (predominantly slow-twitch muscle, myosin heavy chain isoform (MHC) Type I) and *epitrochlearis* (predominantly fast-twitch muscle, MHC Type IIb) [28] were determined using a previously published method [29]. Briefly, after a 4 hours fast, rats were anesthetized with an intraperitoneal injection of sodium pentobarbital (60 mg/kg). After 10–15 minutes, the *epitrochlearis* and *soleus* muscles were carefully removed and rapidly rinsed in PBS. *Soleus* muscles were delicately cut into two longitudinal strips to ensure equal diffusion across the muscle and reduce potential hypoxia. No significant differences were observed between CKD and CTL muscle weights, in both rat strains (Data not shown). Muscles were then pre-incubated in a Krebs-Henseleit buffer (KHB) solution containing 2mM pyruvate and 6mM Mannitol under constant oxygenation (95% O^2^-5% CO^2^) at 35°C. After a 30 min pre-incubation period, muscles were incubated 20 min in KHB containing 1mM of [^3^H]-2-deoxy-D-glucose (2-DG) (American Radiolabeled Chemicals, St. Louis, MO), 9mM [^14^C]-mannitol (American Radiolabeled Chemicals) and 0 or 2mU/ml of insulin (Humulin R, Eli Lilly, Toronto, ON) for SD rats and 0 or 10mU/ml of insulin for Zucker rats. After incubation, muscles were snap frozen in liquid nitrogen. Frozen muscles were weighed, transferred to prechilled glass tissue grinder tubes (Kontes, Vineland, NJ), and homogenized in ice-cold perchloric acid using a glass pestle attached to a motorized homogenizer. 200μl aliquots of supernatants were mixed with 8mL scintillation cocktail (Fisher Scientific, Ottawa, Canada) and used for scintillation counting on a Hidex 300 SL beta counter (Hidex Oy, Turko, Finland) [30].

### Tissue preparation for Western blot analysis

Frozen biopsies of rat liver, kidney, skeletal muscle and fat tissue were homogenized using an overhead stirrer in RIPA buffer (150mM sodium chloride, 1% Triton X-100, 0.5% sodium deoxycholate, 0.1% SDS, 50 mM Tris, pH 8) containing 0.1 mM PMSF at a ratio of 0.2g of tissue per 1mL buffer. Delipidation was performed on adipose tissue homogenate by chloroform extraction [31]. Protein content was determined using the Lowry method [32], using bovine serum albumin as reference protein.

### mRNA expression

Total RNA was extracted from frozen tissue using TRIzol reagent (Invitrogen, Burlington, ON, Canada). RNA from the aqueous fraction was then purified using the RNeasy Mini kit (Qiagen) according to the manufacturer’s protocol including the optional DNA digestion step. One microgram of total RNA was used to prepare cDNA by reverse transcription using the SuperScript VILO cDNA Synthesis kit (Invitrogen). mRNAs encoding for GLUT1 (S*lc2a1*), GLUT2 (S*lc2a2*), GLUT4 (S*lc2a4*), SGLT1 (S*lc5a1*), SGLT2 (S*lc5a2*), GAPDH, β-actin and Villin-1 were measured by quantitative real-time polymerase chain reaction using appropriate primers on an ABI 7500 Real-Time PCR System (Applied Biosystems, Foster city, CA). Taqman Gene Expression Assays used for the quantification of mRNA for each transporter can be found in Table 1. PCR products were analyzed using the ΔΔCT method [33] using GAPDH, β-actin and Villin-1 as housekeeping genes as described above.

**Table 1:** TaqMan gene expression assays used for Real-Time PCR.

	Gene	NCBI Location Chromosome	TaqMan Gene Expression Assay
**Glut1**	*Slc2a1*	Chr.5: 138154677–138182897	Rn01417099_m1
**Glut2**	*Slc2a2*	Chr.2: 114413431–114445418	Rn00563565_m1
**Glut4**	*Slc2a4*	Chr.10: 56552983–56558561	Rn01752377_m1
**Slgt1**	*Slc5a1*	Chr.14: 82910448–82975303	Rn01640634_m1
**Sglt2**	*Slc5a2*	Chr.1: 199682688–199688809	Rn00574917_m1
**GAPDH**	*GAPDH*	Chr.4: 157676396–157680271	Rn01775763_g1
**ActinB**	*actin*, *beta*	Chr.12: 13715843–13718813	Rn00667869_m1
**Villin-1**	*Villin-1*	Chr.9: 81689802–81717623	Rn01254356_g1

### Western blotting

Glucose transporter protein levels were assessed by Western blot analysis. Samples of 5 or 20 μg of protein were separated by electrophoresis on a 9% polyacrylamide gel containing 0.1% sodium dodecyl sulfate (SDS) and were electrophoretically transferred onto Polyvinylidene fluoride (PVDF) membrane. Membranes were saturated with 5% BSA in Tris buffered saline (TBS) containing 0.1% Tween20 (TBST) and washed with TBST. GLUT1 was detected using antibody PA1-1063 (Thermo Scientific Pierce, Rockford, IL), GLUT2 using antibody ARP41706 (Aviva Systems Biology, San Diego, CA), GLUT4 using antibody PA1-1065 (Thermo Scientific Pierce), SGLT1 using antibody Ab-14686 (Abcam, Cambridge, MA), and SGLT2 using antibody AP00335PU-N (Acris Antibodies, San Diego, CA). GAPDH was used as a GLUT4 containing-tissue loading control using antibody Ab9485 (Abcam). β-Actin was used as a loading control for liver samples using antibody GTX23280 (GeneTex, Irvine, CA). Villin-1 was used as a loading control for kidney tissue using antibody R814 (Cell Signaling Technology, Danvers, MA). Antibodies specificities were confirmed with blocking peptides. Specific antibody binding was revealed using appropriate secondary antibodies (Goat anti-rabbit IgG or Goat anti-mouse IgG from Sigma-Aldrich, St. Louis, MO) coupled to peroxidase and with the Clarity Western ECL Substrate from Bio-Rad Laboratories (Hercules, CA). Scanned images are provided in S2 Fig. Results were analyzed by computer-assisted densitometry using ImageQuant LAS-4000 system from GE Healthcare Life Sciences (Mississauga, ON) and FUJIFILM MultiGauge V3.0.

### Statistical analysis

**Fig 1:**
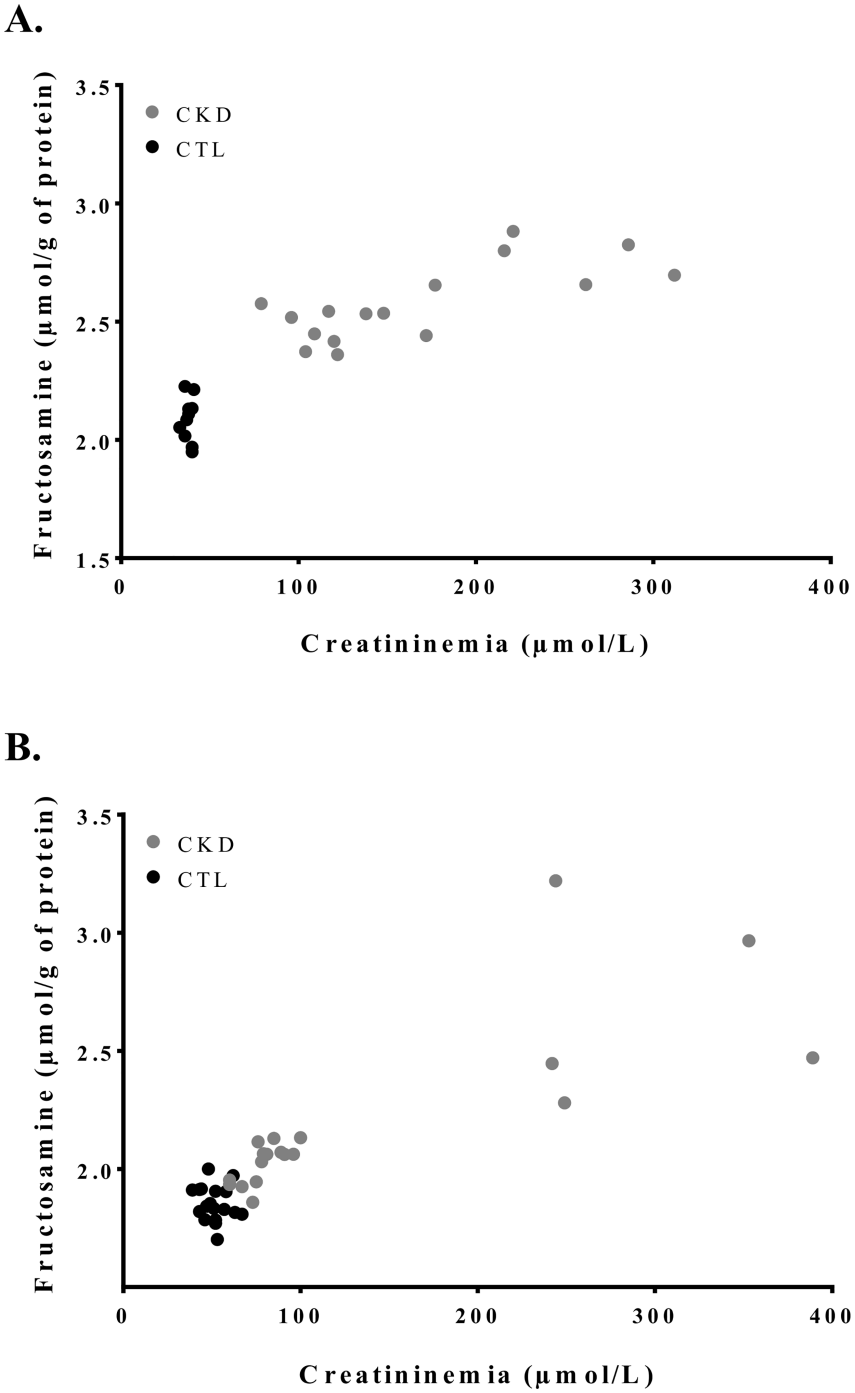
Correlation between serum fructosamine and creatinine in CTL and CKD rats. Fructosamine concentration expressed as a ratio of fructosamine reported on total protein content in serum, versus creatininemia of **(A)** n = 26 SD rats and **(B)** n = 37 Zucker Lepr^fa/fa^ rats. Measurements at Day 21.

Results are expressed as mean ± S.D.. SD rats were analyzed as a cohort using the Student’s t test. Each Zucker CKD rat was analyzed against its own control using a ratio paired Student’s t test. Survival curves were assessed using the Mantel-Cox test. The threshold of significance was p≤0.05. Statistical analyzes were performed with GraphPad Prism v6.05 (La Jolla, California, USA), except for Fig 1, in which non-parametric Spearman correlation analyzes were done with the use of SAS software, version 9.4 (SAS Institute, Cary, NC).

## Results

### Biochemical parameters and body weight of SD rats

As compared to controls, CKD rats had higher levels of serum creatinine and urea and lower creatinine clearance (Table 2). Although glucose levels were not statistically elevated in CKD animals, we nevertheless observed a hyperglycemic state. Indeed, fructosamine levels were 25% higher (p<0.0001) in CKD rats compared to controls. Furthermore, a direct correlation between serum fructosamine and serum creatinine (r_spearman = 0.87, p<0.0001) was observed (Fig 1A). No significant changes in insulinemia were observed.

**Table 2:** Biochemical parameters and body weight of CTL and CKD SD rats at Day 21.

	CTL (n = 17)	CKD (n = 25)	p value
**Body weight (g)**	321.1 ± 10.6	300.7 ± 28.6	˂ 0.01
**Creatininemia (μmol/L)**	40.1 ± 3.8	152.6 ± 66.2	˂ 0.0001
**Uremia (mmol/L)**	3.2 ± 0.8	23.0 ± 11.4	˂ 0.0001
**Creatinine clearance (μl/100g b.wt./min)**	527.8 ± 96.9	111.8 ± 50.7	˂ 0.0001
**Glycemia (mmol/L)**	6.0 ± 1.0	6.5 ± 1.2	N.S.
**Insulinemia (ng/mL)**	1.05 ± 0.51	0.91 ± 0.54	N.S.
**Fructosamine (μmol/g of protein)**	2.09 ± 0.09	2.58 ± 0.16	< 0.0001

### Intraperitoneal glucose tolerance test on SD rats

**Fig 2:**
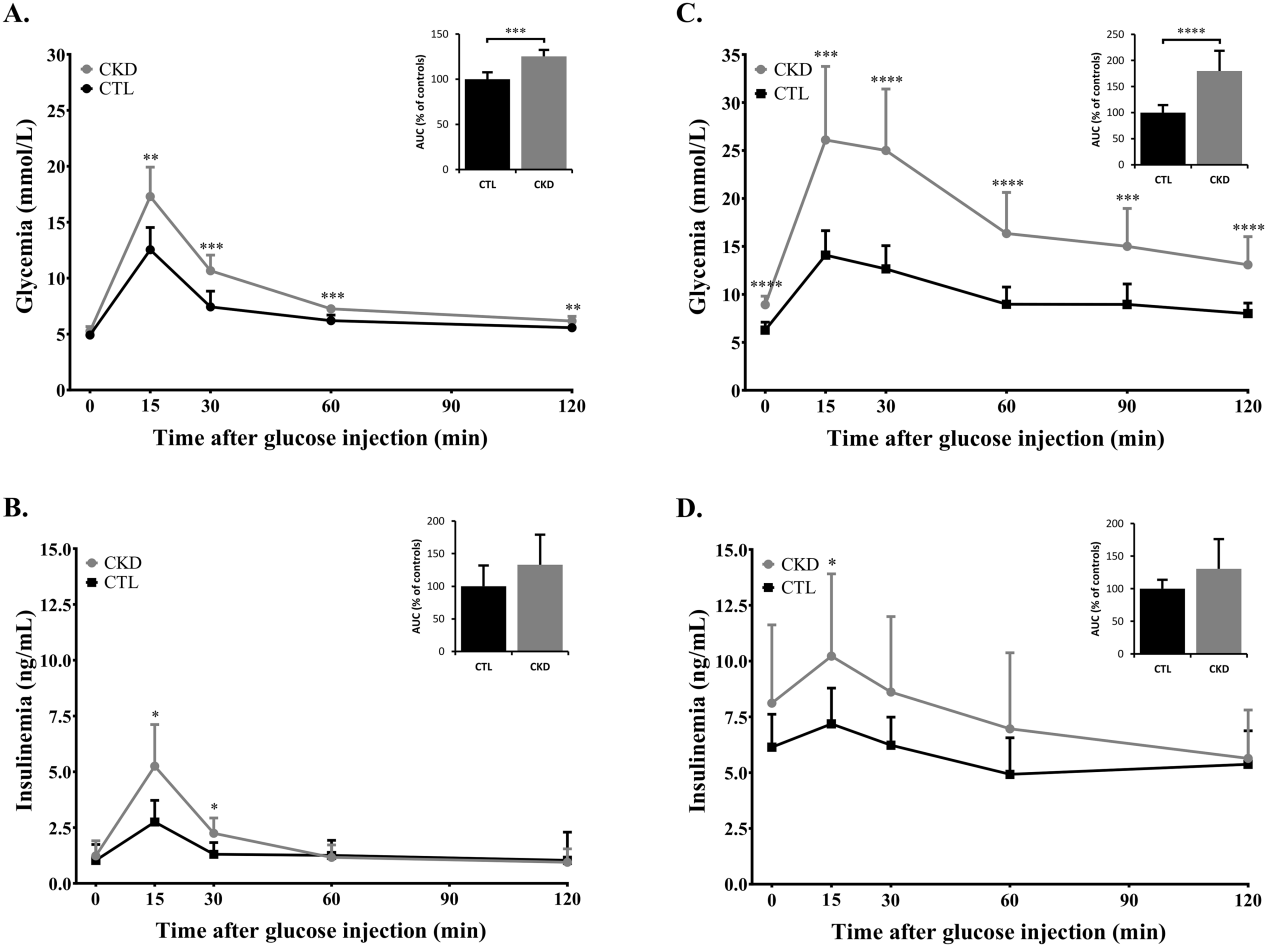
Glucose and insulin responses to an intraperitoneal glucose tolerance test. **(A)** Glucose and **(B)** Insulin responses to an intraperitoneal glucose tolerance test (2g/kg) performed on SD rats at Day 28 following a 16 hours fast. **(C)** Glucose and **(D)** Insulin responses to an intraperitoneal glucose tolerance test (1.5g/kg) performed on Zucker Lepr^fa/fa^ rats at Day 28 following a 16 hour fast. Values are mean ± S.D. n = 10 for each group. *, p<0.05; **, p<0.01; ***, p<0.001; ****, p<0.0001 as compared to CTL rats. Area under the curve is presented as percentage of controls, on the right corner of each graph.

A glucose tolerance test (GTT) performed on SD rats at 4 weeks post nephrectomy (Day 28) resulted in impaired carbohydrate metabolism in the nephrectomised cohort, as indicated by higher glycemic levels at each time-point studied, in comparison to control rats (Fig 2A). Glucose-stimulated insulin secretion (GSIS) was also impaired in nephrectomised rats, as demonstrated by a 30% greater insulin AUC over a 2 hours period, compared to control rats (Fig 2B).

### mRNA expression of SGLT and GLUT glucose transporters in SD rats

**Fig 3:**
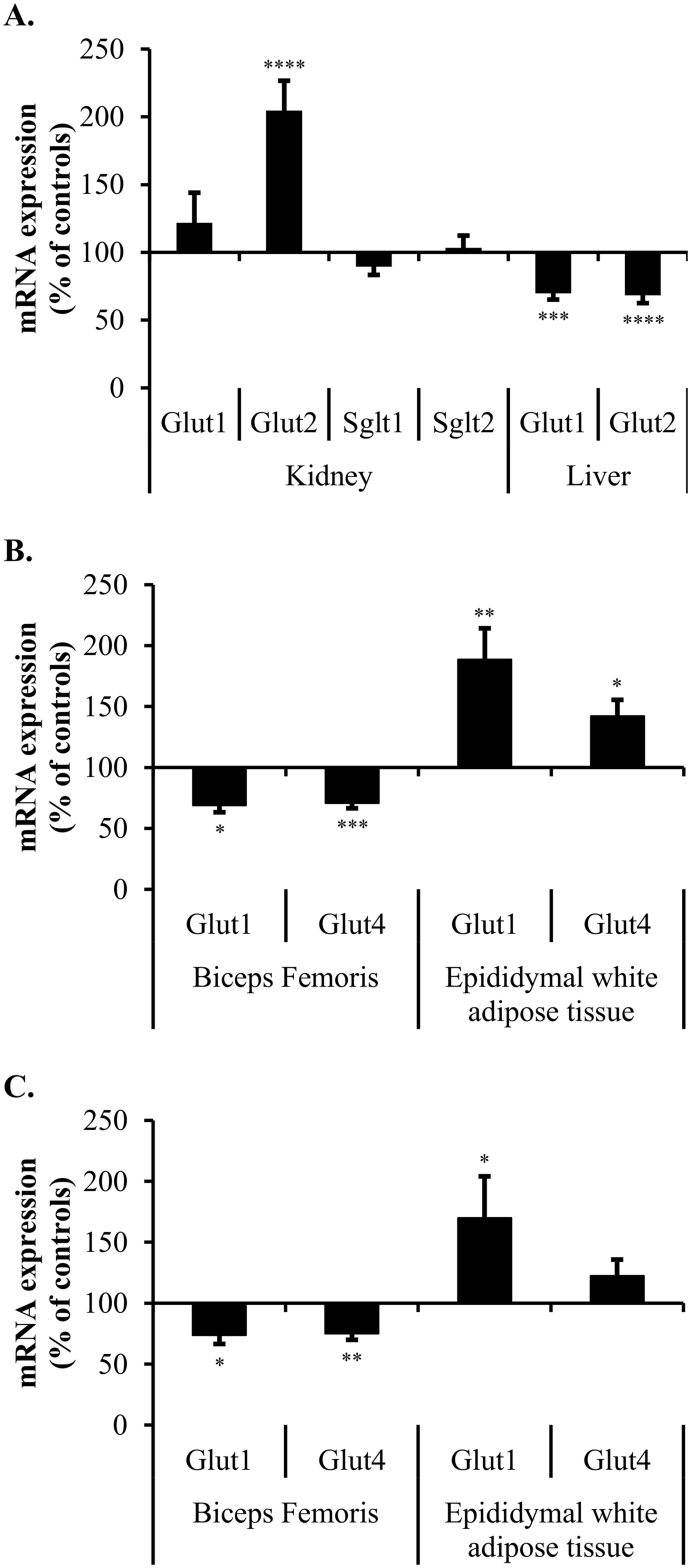
Glucose transporters mRNA expression in selected organs of CKD rats. mRNA encoding glucose transporters in CTL and CKD rats in the indicated tissues were measured by quantitative Real-Time PCR. mRNA levels are expressed in relative quantities and calculated using the ΔΔCT method [33] with their respective housekeeping gene (Villin-1 for kidneys, β-actin for liver and GAPDH for muscles and adipose tissues). Data was normalized to the mean relative quantity of each gene in CTL rats. The graph shows the mean expression in CKD rats expressed as a percentage of controls ± S.D. of at least 10 rats in each group. *, p<0.05; **, p<0.01; ***, p<0.001; ****, p<0.0001 as compared to CTL rats. **(A)** Glucose transporters mRNA expression in the kidney and liver of SD rats. **(B)** GLUT1 and GLUT4 mRNA expression in skeletal muscle and white adipose tissue of SD rats. **(C)** GLUT1 and GLUT4 mRNA expression in skeletal muscle and white adipose tissue of Zucker Lepr^fa/fa^ rats. Measurements at Day 42.

Analysis of mRNA encoding glucose transporters involved in carbohydrate homeostasis in key organs is presented in Fig 3. There was a significant decrease in the expression of GLUT1 and GLUT2 (decrease of 31% and 32%, p<0.001 and p<0.0001 respectively) in CKD rat livers as compared to controls. In addition, renal impairment induced an increase in kidney GLUT2 expression (increase of 105%, p<0.0001) compared to control rats. However, mRNA expression of GLUT1, SLGT1 and SGLT2 in the kidney remained stable. Both GLUT1 and GLUT4 mRNA expression in muscle were decreased by CKD (decrease of 31% and 29%, p<0.05 and p<0.001 respectively). GLUT1 and GLUT4 mRNA expression levels in the adipose tissue were increased (increase of 89% and 42%, p<0.01 and p<0.05 respectively).

### Protein levels of GLUT and SGLT glucose transporters in SD rats

**Fig 4:**
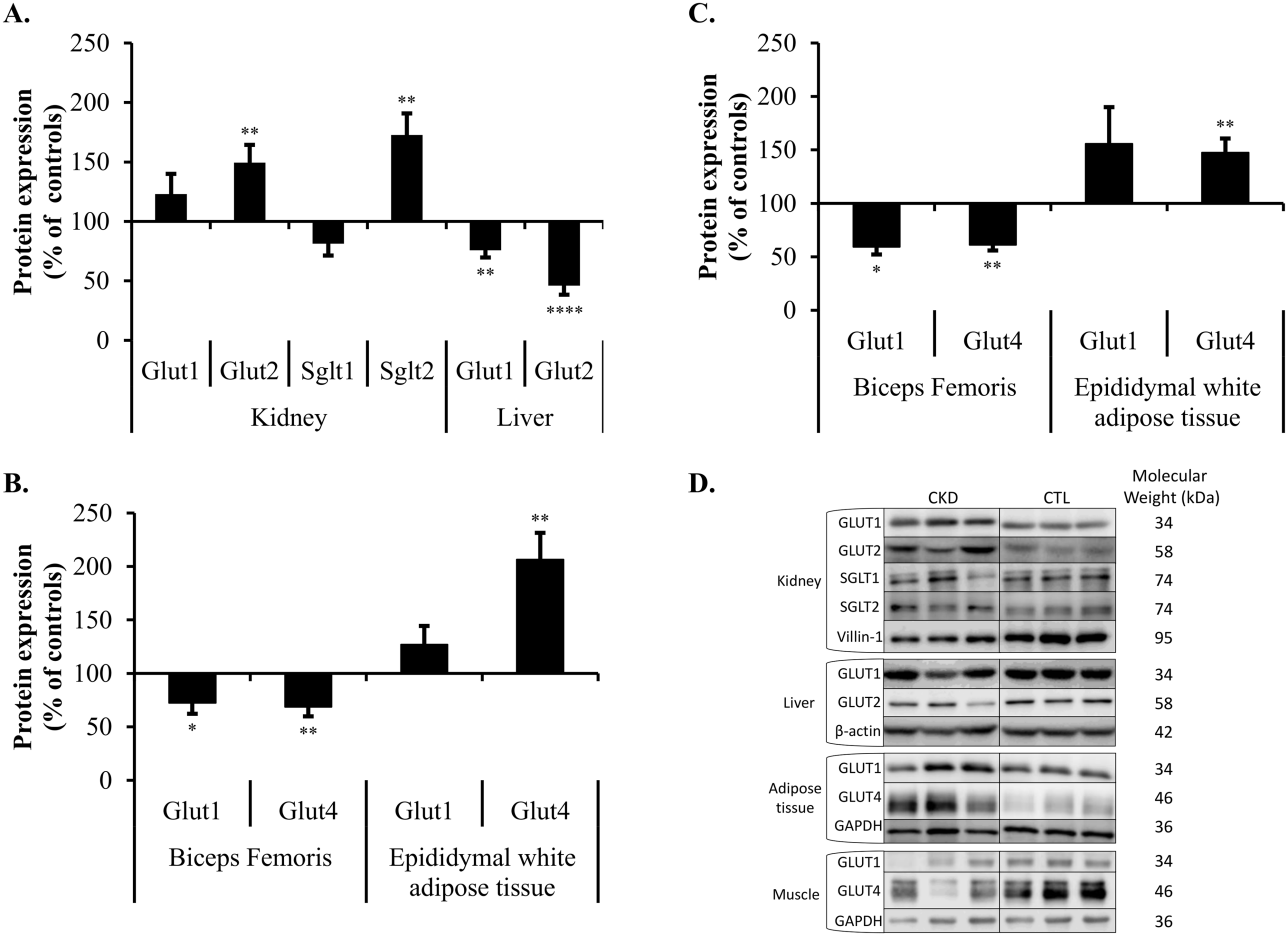
Glucose transporters protein expression in selected organs of CKD rats. Protein levels are expressed in densitometry units. The densitometry units measured for glucose transporters were normalized to their respective loading control (β-actin: liver; GAPDH: muscles and adipose tissues; villin-1: kidneys) values. The normalized densitometry units of control rats were arbitrarily defined as 100%. The graph shows the mean expression in CKD rats expressed as percentage of CTL ± S.D of at least 5 rats in each group. *, p<0.05; **, p<0.01; ***, p<0.001; ****, p<0.0001 as compared to CTL rats. Protein levels of glucose transporters in the **(A)** Kidneys and liver of SD rats, **(B)** Skeletal muscle and white adipose tissue of SD rats. **(C)**. Skeletal muscle and white adipose tissue in Zucker Lepr^fa/fa^ rats. **(D)**. Representative Western blots for each glucose transporter. Each blot contains examples of three CKD (left) and three CTL (right) SD rats. Measurements at Day 42.

Fig 4 presents the relative protein levels of the principal glucose transporters in various tissues involved in carbohydrate metabolism. Renal impairment caused a down-regulation of hepatic GLUT1 and GLUT2 (decrease of 24% and 54%, p<0.01 and p<0.0001 respectively) as compared to control rats. Conversely, kidney GLUT2 and SGLT2 levels were increased in the presence of CKD (increase of 49% and 73%, p<0.01 and p<0.01 respectively). Kidney levels of GLUT1 and SGLT1 remained unchanged. In muscle, both GLUT1 and GLUT4 were considerably diminished (decrease of 27% and 31%, p<0.05 and p<0.01 respectively) by kidney disease, and GLUT1 and GLUT4 were inversely affected by CKD in adipose tissue as compared to the muscle (increase of 27% and 106%, N.S. and p<0.01 respectively).

### *Ex Vivo* uptake of radiolabeled 2-deoxyglucose in the *epitrochleari*s and the *soleus* muscles of SD rats

**Fig 5:**
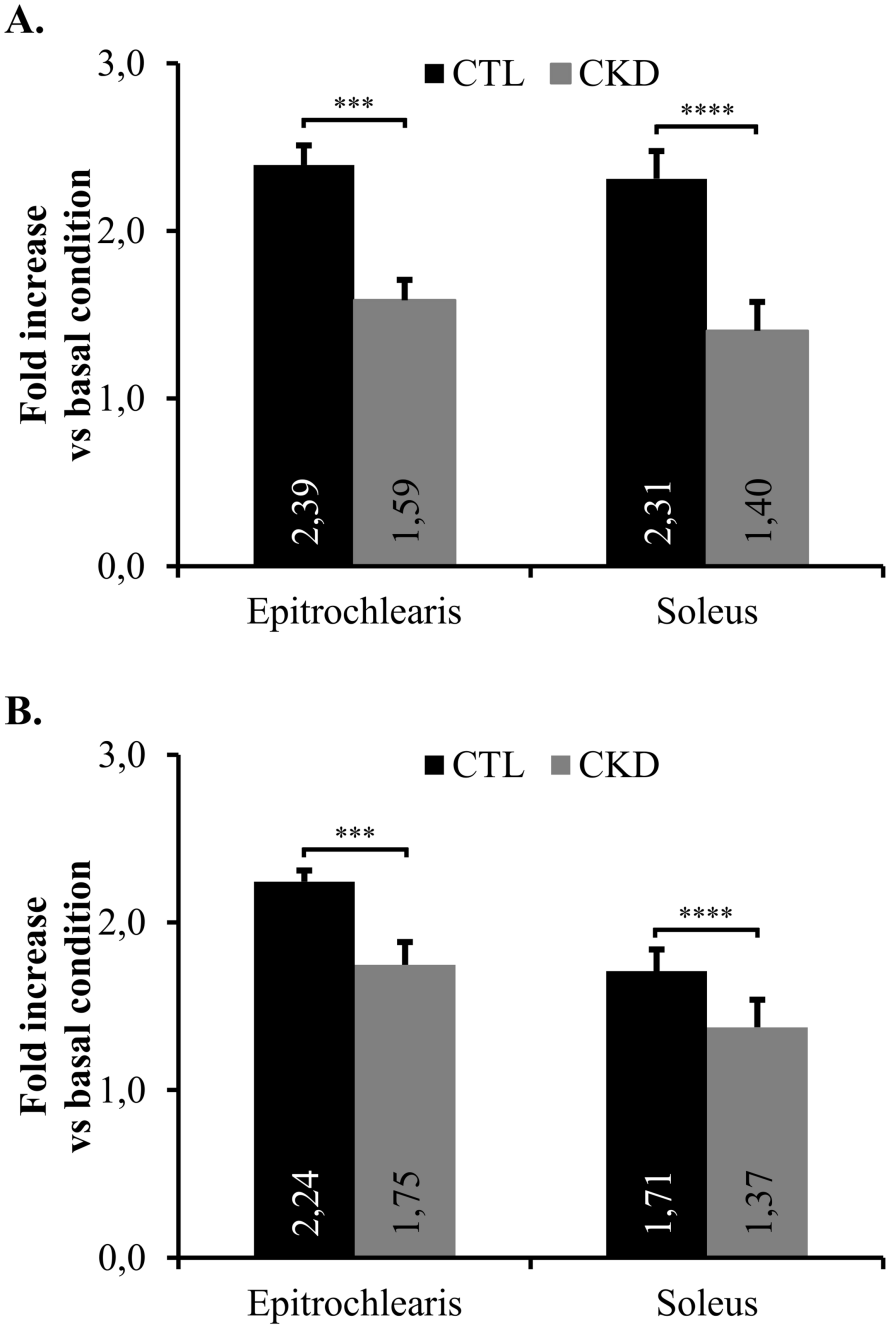
*Ex vivo* accumulation of radio-labeled 2-deoxyglucose in muscles. Uptake of radio-labeled 2-deoxyglucose in CTL and CKD rat muscles expressed as insulin response vs. basal condition. Basal condition of each muscle was arbitrarily defined as 1,0. Data are expressed as fold increase (mean) ± S.D for at least 5 rats per group. ***, p<0.001; ****, p<0.0001 as compared to CTL rats. **(A)** Sprague-Dawley rats and **(B)** Zucker Lepr^fa/fa^ rats. Measurements at Day 42.

Analysis of insulin stimulated glucose uptake (measured by fold increase in glucose uptake) revealed a lower glucose uptake in the CKD cohort for both *epitrochlearis* (decrease of 34% vs controls, p<0.0001) and *soleus* (decrease of 39% vs controls, p<0.0001) muscles (Fig 5A). Interestingly, as shown in supporting information S3A Fig, we observe that insulin induced a greater glucose uptake in the *soleus* than in the *epitrochlearis*. These findings are in accordance with the results of Henriksen *et al*. [34] pointing out the importance of fiber type selective differences in glucose uptake [35].

### Insulin resistance in Zucker rats is affected by CKD

Similar to SD rats, CKD Zucker rats showed an increase in serum fructosamine that correlated with serum creatinine levels (Fig 1B) (r_spearman = 0.78, p<0.0001). Unlike SD rats, Zucker rats with CKD had an elevated fasting glycemia (increase of 50% vs controls, p<0.0001) without higher insulin levels (Table 3).

**Table 3:** Biochemical parameters and body weight of CTL and CKD Zucker Lepr^fa/fa^ rats (Day 21).

	CTL (n = 19)	CKD (n = 19)	p value
**Body weight (g)**	345.6 ± 13.3	340.8 ± 13.4	N.S.
**Creatininemia (μmol/L)**	51.8 ± 1.7	136.2 ± 23.8	˂ 0.0001
**Uremia (mmol/L)**	5.5 ± 0.3	29.6 ± 5.4	˂ 0.0001
**Creatinine clearance (μl/100g b.wt./min)**	146.3 ± 7.1	83.2 ± 9.2	˂ 0.01
**Glycemia (mmol/L)**	6.0 ± 0.2	9.1 ± 0.6	˂ 0.0001
**Insulinemia (ng/mL)**	5.76 ± 0.92	6.65 ± 0.85	N.S.
**Fructosamine (μmol/g of protein)**	1.85 ± 0.02	2.20 ± 0.08	< 0.0001

Insulin resistant Zucker rats showed an even greater response to glucose load under CKD conditions (Fig 2C and 2D) as compared to SD rats, and demonstrated a similar impairment in insulin levels. In addition, changes in glucose transporter mRNA expression (Fig 3C) in CTL and CKD Zucker rats were similar to that observed in SD rats in the muscle (GLUT1 decrease of 26%, p<0.05; GLUT4 decrease of 25%, p<0.01) and adipose tissue (GLUT1 increase of 70%, p<0.05; GLUT4 increase of 23%, N.S.). This was also true of glucose transporter protein levels of Zucker CTL versus CKD rats (Fig 4C) with a reduction of glucose transporters in muscles (GLUT1 decrease of 41%, p<0.01; GLUT4 decrease of 39%, p<0.01) and higher protein levels in adipose tissues (GLUT1 increase of 56%, p<0.05; GLUT4 increase of 47%, N.S.).

Differences in insulin stimulated glucose uptake in muscles from Zucker CTL and CKD rats were also observed. Since 2mU/mL of insulin did not significantly increase glucose uptake by muscles from Zucker rats with CKD (data not shown), a higher dose of insulin (10mU/mL) was necessary to induce insulin-stimulated glucose uptake in the muscles of these rats (Fig 5B). As compared to controls, Zucker rats affected by CKD had a reduced insulin stimulated glucose uptake in both *epitrochlearis* (decrease of 22% vs controls, p>0.001) and *soleus* (decrease of 20% vs controls, p>0.0001) muscles. As observed in supporting information S3B Fig, in Zucker rats, insulin induced a greater glucose uptake in the *soleus* than in the *epitrochlearis*. This difference was slightly less than in SD rats.

### Presence of glucose in urine (Glycosuria)

**Fig 6:**
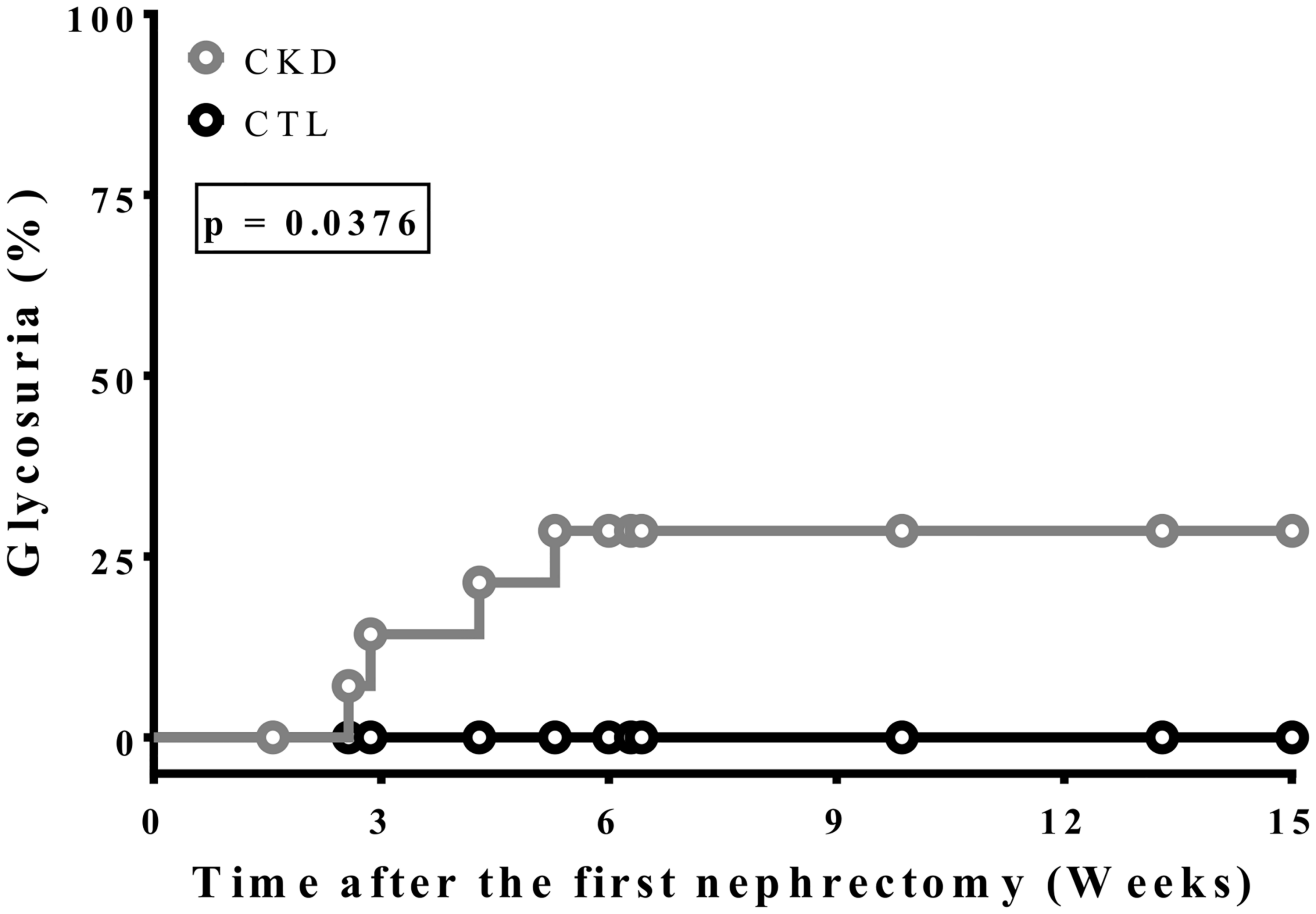
Time of glycosuria apparition in Zucker Lepr^fa/fa^ rats. The graph shows the proportion of rats with glycosuria as confirmed by a positive Diastix^™^ (Bayer) test on two consecutive days for 15 Zucker Lepr^fa/fa^ rats per group.

The presence of glycosuria is due to the inability of the kidney to reabsorb filtered glucose or spillage due to abnormally high levels of blood glucose. Spillage does not generally occur until blood glucose exceeds ~10mmol/L [36]. None of the SD rats had detectable urine glucose levels. In contrast, ~30% of the Zucker rats with severe CKD (creatininemia ≥ 200μmol/L) presented glycosuria whereas none in the control Zucker rats had detectable urine glucose (Fig 6).

## Discussion

Despite extensive study of glucose transporters, there is little and conflicting information regarding their role in CKD. Our results suggest that in a 5/6^th^ nephrectomised Sprague-Dawley rat model, there is a correlation between the severity of chronic kidney disease and hyperglycemia as evaluated by serum fructosamine levels. Glucose tolerance tests also indicate an increase of 25% in glycemia in chronic kidney disease rats as compared to controls, whereas insulin levels remained unchanged. We also observed modulation of glucose transporters expression in several tissues such as the liver and muscles. There was a significant reduction in insulin-dependent glucose uptake in the muscles of chronic kidney disease rats but the development of type 2 diabetes was not observed. Furthermore, in a rat model of metabolic syndrome (Zucker Lepr^fa/fa^), chronic kidney disease caused a significant increase in fasting glucose and an exacerbated glycemic response during glucose challenge. Similar modulations of glucose transporters expression and glucose uptake were observed in the two models. However, 30% of chronic kidney disease Zucker rats developed characteristics of type 2 diabetes.

In the present study, we provided evidence that CKD rats have chronic hyperglycemia as indicated by a significant increase in serum fructosamine, although fasting glycemia was not significantly elevated. To further characterize the insulin sensitivity and secretion in our models of CKD, we performed glucose tolerance tests (GTT). Our results confirmed impaired glucose tolerance in the nephrectomised cohort as revealed by their inability to restore basal blood glucose levels (Fig 2A) despite increased insulin levels (Fig 2B). The increased insulin concentration was insufficient to counteract hyperglycemia as higher blood glucose levels were measured in rats with renal impairment, thus providing evidence of an insulin resistance evolution.

Having established that CKD may lead to insulin resistance, the expression of glucose transporters was measured in several key organs involved in glucose metabolism, in order to determine the mechanisms of this metabolic condition when kidney function is compromised. We primarily focused on fat and muscle glucose transporters, as impaired insulin sensitivity in CKD is generally ascribed in peripheral tissues [37, 38]. Furthermore, we focused on GLUT4, as any abnormality affecting its expression, its translocation and/or its activity has been strongly linked to insulin resistance [39, 40]. Interestingly, our results show a significant decrease in GLUT4 protein levels in skeletal muscle from rats with impaired renal function (Fig 4).

Glucose uptake studies were performed on skeletal muscle since this tissue represents the main site of insulin resistance in CKD [37, 41]. As skeletal muscle glucose transport can be stimulated by both insulin and contractile activity [34], we used sedentary rats to measure the glucose transport mediated principally by insulin. Experiments were done *ex vivo* to avoid any extra-muscular hormonal and metabolic factors that may influence glucose uptake [42, 43]. Our results demonstrated a clear reduction of insulin-stimulated glucose transport in CKD muscles (Fig 5) which is likely attributable, at least in part, by the down-regulation of muscle GLUT4 glucose transporter expression in kidney failure. Friedman and colleagues have already reported lower glucose uptake by skeletal muscle preparations obtained from uremic patients while the expression of GLUT4 remained unchanged [17], whereas Aksentijevic and colleagues noted diminished GLUT4 protein expression in the skeletal muscles of uremic animals [21]. Furthermore, insulin acts on microvascular perfusion to promote glucose uptake in peripheral tissues. The impact of kidney disease on insulin-mediated microvascular responses has not been studied and may contribute to a reduction in glucose uptake in the muscles under *in vivo* conditions [44, 45]. Thus, the down-regulation of GLUT4 expression in skeletal muscle of nephrectomised rats may explain the insulin resistance in kidney disease. That said, other organs are also involved in carbohydrate metabolism and may contribute to the insulin resistance.

The presence of defective hepatic glucose metabolism in CKD is often disregarded, and no study has focused on the modulation of liver glucose transporters in CKD. Notably, our results demonstrate a substantial decrease in the protein levels of GLUT1 and GLUT2 (Fig 4A). As the liver extracts approximately 1/3 of oral glucose loads, a decrease of glucose transporters in this tissue could have major repercussions on blood glucose regulation [46]. Basu *et al*. have suggested that impaired glucose uptake in the liver and peripheral tissues could contribute to hyperglycemia in people with T2D [47]. However, few studies have suggested impaired hepatic glucose disposal and production as a cause of insulin resistance in CKD. According to Schmitz *et al*., uremia seems to clearly impair glucose-induced glucose uptake by the liver [48].

The kidneys play an important role in glucose homeostasis as they ensure its reabsorption through glucose transporters. The modulation of glucose transporters in renal impairment has rarely been mentioned in the literature. Our data suggest upregulation of kidney GLUT2 and SGLT2 levels in CKD (Fig 4A). Since GLUT2 and SGLT2 transporters are responsible for ~90% of glucose reabsorption, this implies that the kidneys try to reabsorb more glucose from the glomerular filtrate. In a rodent model of T2D with long-term hyperglycemia, kidney GLUT2 and SGLT2 levels are increased. Thus, it seems plausible that dysregulation of GLUT2 and SGLT2 could contribute to the hyperglycemia in CKD [49].

We have not observed any case of T2D among SD rats with kidney disease. Since CKD commonly arises alongside other metabolic imbalances such as metabolic syndrome, kidney disease could worsen glucose homeostasis in these rats. The Zucker Lepr^fa/fa^ is a rat model affected by insulin resistance and metabolic syndrome. Inducing kidney disease in Zucker rats exacerbated the hyperglycemia state (Fig 1B) and insulin resistance (Fig 2B and 2D) as compared to SD rats. The modulation of glucose transporters were similar (Figs 3C and 4C) to those observed in SD rats, but the reduction in glucose uptake by the muscle was more severe (Fig 5B). Kidney disease and metabolic syndrome in Zucker rats acts synergistically on glucose homeostasis and reduced insulin sensitivity. Moreover, glycosuria appeared in severely affected CKD Zucker rats with a fasting glycemia near 11mmol/L. Glycemia rose to a critical point at which the kidneys can no longer entirely reabsorb the filtered glucose. Thus, these rats displayed a chronic hyperglycemic state, which can be associated with T2D as insulinemia did not rise to normalize blood glucose [50].

Some limitations of this study should be mentioned. First, although we measured circulating insulin levels, the insulin secretion of CKD rats was not directly measured *in vitro*. Indeed, Koppe *et al*. [10] recently showed that urea caused impaired insulin secretion in pancreatic β-cells. Whether this mechanism could partly explain our results remains to be confirmed. Second, since reduced translocation to the plasma membrane can only be presumed from total GLUT4 protein levels, this issue will have to be further investigated. Third, it remains to be demonstrated if our results apply to CKD patients. A recent study by de Boer *et al*. [51] has showed that in moderate CKD patients (mean eGRF 37 mL/min per 1.73 m^2^,) 65% of them have impaired glucose tolerance and there was no association of kidney function with insulin resistance parameters. Others parameters such as lifestyles factors or body composition have been proposed to partially explain the insulin resistance in these patients. However, in the current study, these factors have been controlled. Furthermore, it does not exclude the hypothesis that as renal failure worsens (in our CKD rats, the GFR was reduced by 80%, which means stage 4 and 5 in humans), CKD plays a direct role in insulin resistance. More importantly, our results emphasize that in predialysis patients, insulin resistance, and possibly T2D development, should be followed more closely.

In conclusion, GTT experiments showed an altered glucose transporter expression that could be associated with an impairment of glucose metabolism in CKD. Indeed, a significant reduction of GLUT4 in skeletal muscle was likely important for the measured insulin resistance. This modulation may also explain the reduced glucose uptake by muscles reported in CKD. Moreover, the increased protein expression of the majority of kidney glucose transporters in CKD may allow greater glucose reabsorption while hyperglycemia further contributes to the exacerbation of insulin resistance. Reduced levels of hepatic GLUT1 and GLUT2 proteins may reflect dysregulated glucose metabolism by the liver in CKD and may suggest the presence of hepatic glucose intolerance that would subsequently contribute to the impaired glucose metabolism in CKD. Thus, the insulin resistance seen in CKD could be mediated, in part, by the modulation of the expression of various key transporters involved in glucose homeostasis. However, CKD by itself does not lead to T2D. Rather, CKD seems to worsen the insulin resistance in Zucker rats in which insulin resistance is already present before nephrectomy.

## Supporting information

S1 Fig Timeline of experimentation. (TIF)

S2 Fig Western blots. (ZIP)

S3 Fig *Ex vivo* accumulation of radio-labeled 2-deoxyglucose in muscles. Uptake of radio-labeled 2-deoxyglucose in CTL and CKD rat muscles. Data are expressed as 2-DG uptake (mean) ± S.D. for **(A)** 2 representative experiments in Sprague-Dawley rats and **(B)** 3 representative experiments in Zucker Lepr^fa/fa^ rats.
(TIF)
