# Supplementary figures and images for: Mechanism of insulin resistance in a rat model of kidney disease and the risk of developing type 2 diabetes
Source: PLoS One. 2017 May 1;12(5):e0176650. doi: 10.1371/journal.pone.0176650 (PMC5411038; doi:10.1371/journal.pone.0176650)

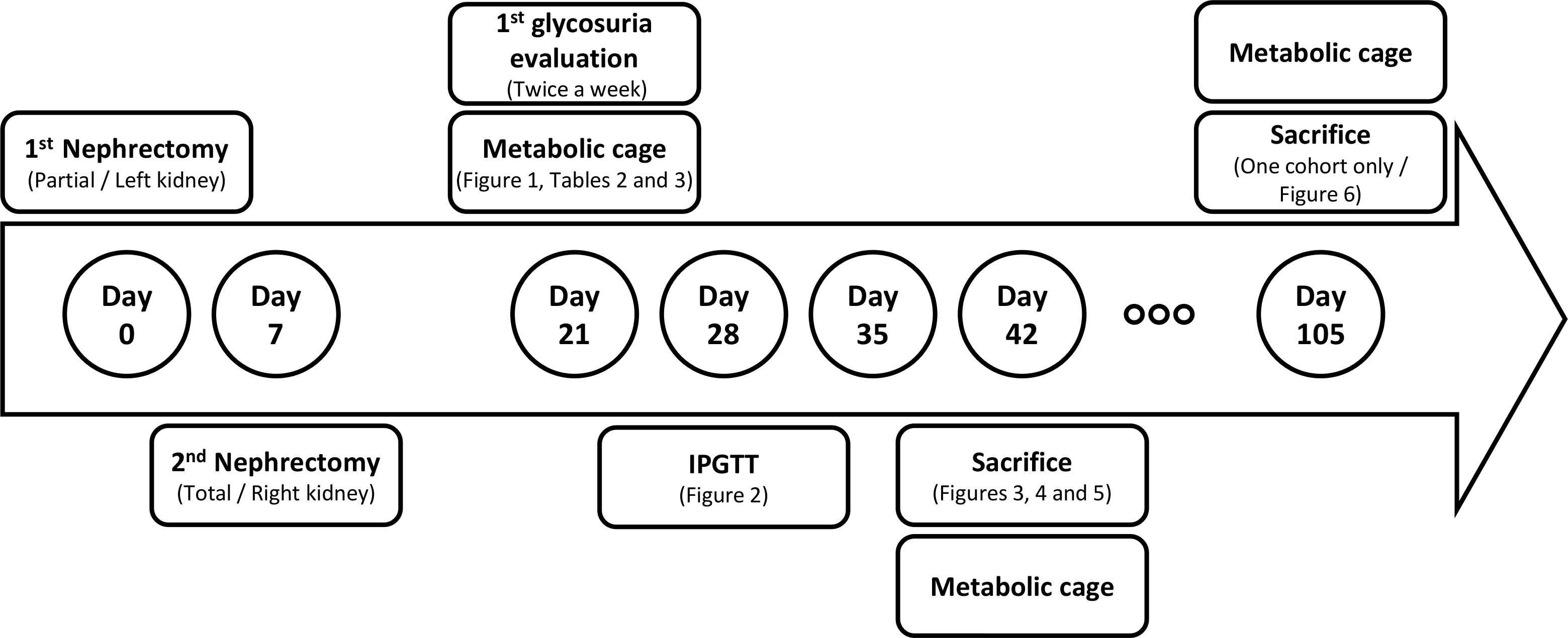

Supplement: S1 Fig — (TIF) [file pone.0176650.s001.tif]

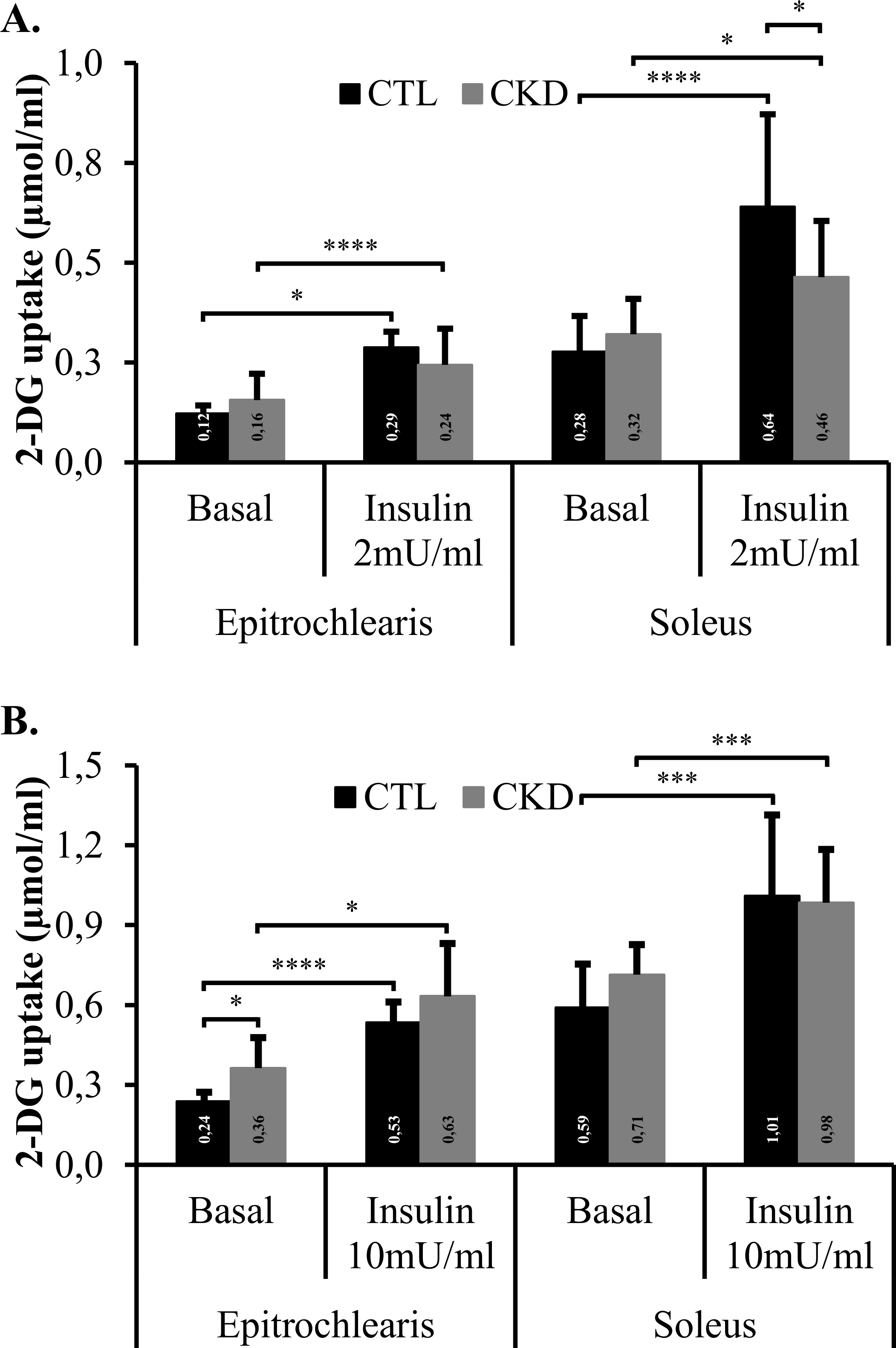

Supplement: S3 Fig — Uptake of radio-labeled 2-deoxyglucose in CTL and CKD rat muscles. Data are expressed as 2-DG uptake (mean) ± S.D. for (A) 2 representative experiments in Sprague-Dawley rats and (B) 3 representative experiments in Zucker Leprfa/fa rats. (TIF) [file pone.0176650.s003.tif]
